# Incidental versus symptomatic nonfunctioning pituitary adenomas: Are they different?

**DOI:** 10.1002/edm2.445

**Published:** 2023-09-11

**Authors:** Vanessa Guerreiro, Fernando Mendonça, Helena Urbano Ferreira, Paula Freitas, Josué Pereira, Irene Bernardes, Jorge Pinheiro, Tiago Guimarães, Davide Carvalho

**Affiliations:** ^1^ Department of Endocrinology, Diabetes and Metabolism Centro Hospitalar São João, EPE Porto Portugal; ^2^ Faculty of Medicine of the University of Porto Porto Portugal; ^3^ Instituto de Investigação e Inovação em Saúde University of Porto Porto Portugal; ^4^ Department of Neurosurgery Centro Hospitalar de São João, EPE Porto Portugal; ^5^ Department of Neuroradiology Centro Hospitalar de São João, EPE Porto Portugal; ^6^ Department of Anatomic Pathology Centro Hospitalar de São João, EPE Porto Portugal; ^7^ Department of Clinical Pathology Centro Hospitalar de São João, EPE Porto Portugal; ^8^ EPIUnit, Instituto de Saúde Pública Universidade do Porto Porto Portugal

**Keywords:** adenomas, hypopituitarism, nonfunctioning, pituitary

## Abstract

**Background:**

Nonfunctioning pituitary adenomas (NFPAs) constitute one of the most common tumours in the sellar region and are often discovered only when associated with compressive symptoms. With the frequent use of brain imaging, there has been an increase in the prevalence of incidentally discovered NFPAs.

**Aim:**

We aim to determine the prevalence of incidental diagnosis with NPAs observed over a decade and compare the analytical, clinical and treatment differences between those who were diagnosed either incidentally or symptomatically. We also intend to evaluate the pathology differences between both groups.

**Methods:**

We retrospectively analysed patients aged ≥18 years with an apparent NFPA, defined as a pituitary lesion compatible with pituitary adenoma which is not associated with the clinical or biochemical evidence of a hormone‐secreting tumour. Inclusion criteria included normal prolactin level for lesions <9 mm or a prolactin level <100 ng/mL for lesions ≥10 mm in maximal tumour diameter.

**Results:**

We included 119 patients [53.8% males; mean age: 56.8 years (SD = 16.7)]. Diagnosis was incidental in 47.1% of patients, and many patients had unappreciated signs and symptoms of pituitary disease. In the symptomatic and incidental groups, 66.7% and 41.1% of patients had hypopituitarism, respectively (*p* = .005). Only 20.4% of patients incidentally diagnosed had microadenoma (*p* = .060). Hypopituitarism was present in 18.8% of those patients with microadenomas. Most tumours were macroadenomas (87.4%). Half of those patients diagnosed incidentally were submitted to surgery, compared with 75.8% of those who were diagnosed symptomatically (*p* = .004).

**Conclusions:**

Nonfunctioning pituitary adenomas are commonly diagnosed incidentally, with many manifesting symptoms on examination. NFPAs incidentally diagnosed are more commonly macroadenomas and less frequently associated with hypopituitarism than symptomatic. Accordingly, if there was a greater level of knowledge and more suspicion about these pathologies, it might be possible to discover them earlier.

## Graphical abstract

Nonfunctioning pituitary adenomas are commonly diagnosed incidentally, with many manifesting symptoms on examination. If there was a greater level of knowledge and more suspicion about these pathologies, it might be possible to discover them earlier.

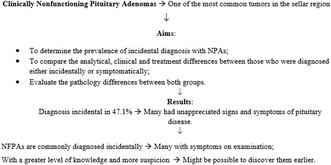

## 1 INTRODUCTION

Pituitary adenomas or, as more recently proposed, ‘pituitary neuroendocrine tumours’ (PitNETs)^1^ are the most common type of tumours found in the pituitary gland.^2^ Nonfunctioning pituitary adenoma (NFPA) benign neoplasms that originate from the adeno hypophyseal cells are diagnosed in the absence of clinical and biochemical evidence of hormonal hypersecretion.^3^

Traditionally, due to the absence of clinical manifestations of hormonal hypersecretion in NFPAs, there is a significant delay in their diagnosis and, therefore NFPAs remain frequently undiagnosed until they become large enough to cause symptoms due to mass effect on surrounding structures.^4^ As a result, many NFPA studies are surgical series.^5^, ^6^ Less common is when these tumours are incidentally diagnosed. Nowadays, with the widespread use of sensitive brain imaging techniques, the incidental detection of pituitary lesions (pituitary incidentaloma [PI]) in images of the head performed by unrelated indications has increased PI diagnosis.^7^ As there are few contemporary studies^8^, ^9^ that analysed these tumours, especially including both patients undergoing surgery as with conservative follow‐up, nowadays the true prevalence of incidentally diagnosed NFPAs is not yet known, neither is their differences in relation to those that are symptomatically diagnosed, as well as the extent to which they possessed previously unrecognized abnormalities. Furthermore, to the best of the authors' knowledge, only four studies have been carried out,^9^, ^10^, ^11^, ^12^ that compare outcomes between incidentally or symptomatically diagnosed adenomas, and only two has previously been carried out with patients with nonfunctioning adenomas only.^9^, ^11^ Of those two studies only one evaluated the pathological differences between the two groups but it included only tumours that underwent surgical resection.^11^

It is essential to extend the frontier of knowledge in this area to ensure that the clinical approach towards certain signs and symptoms and also the discovery of masses in the pituitary gland adapts accordingly. In this sense, we carried out a retrospective observational study of patients observed with NFPA at our hospital over a decade, including patients whose treatment was surgery and also those who remained under clinical surveillance.

We analysed the patients observed with NFPAs in our hospital, in order to compare the analytical, clinical and treatment differences between those who were incidentally or symptomatically diagnosed. We also sought to evaluate the pathological differences between both groups.

## 2 METHODS

### 2.1 Study design and participants

A retrospective observational study was performed on patients aged 18 years of age or more, who were diagnosed at our hospital between 2010 and 2020 with an apparent NFPA, defined as a pituitary lesion that is more consistent with a pituitary adenoma on imaging, without evidence of a clinical or biochemical hormone‐secreting tumour.

The presence of a lesion in the pituitary fossa with or without bone enlargement, which may have an extra‐stellar extension and with imaging characteristics of adenoma was considered to be compatible with a pituitary adenoma.

Initial hormonal screening was based on a general physical examination and symptomatology following the Endocrine Society's guidelines.^13^

Of the 129 patients assessed with NFPAs at our hospital during this period, 10 were excluded due to the absence of analytical study or preoperative symptoms, leading to a final sample of 119 patients. Only 32.8% of patients were referred by neurosurgeons.

All the procedures performed in the study were approved by the Ethics Committee for Health of Centro Hospitalar Universitário de São João. Formal consent is not required for this type of study, in accordance with the national legislation and the institutional requirements.

### 2.2 Data collection

Data were retrieved from the clinical files. During the first medical appointment, all patients underwent a comprehensive clinical history (including questioning about signs or symptoms potentially related to tumour mass effect or hypopituitarism), as well as a physical examination. We evaluated how the patients' pituitary tumour came to medical attention and they were accordingly categorized as being either incidentally diagnosed (i.e. the discovery of a pituitary lesion during imaging carried out for a reason other than suspected pituitary disease), or symptomatically related to the tumour.

Data on the morning peripheral blood sampling were also collected for the measurement of cortisol, prolactin, free thyroxine (FT4), thyroid‐stimulating hormone (TSH), growth hormone (GH), IGF‐1, luteinizing hormone (LH), follicle‐stimulating hormone (FSH) and oestradiol or testosterone in women and men, respectively.

Hormones were measured by chemiluminescent immunometric assay from IMMULITE 2000® (IGF‐1, GH) or ARCHITECT® (TSH, FT4), or by electrochemiluminescence from COBAS e411® (cortisol, FSH, LH, oestradiol and testosterone) and the results were compared with the manufacturers' established reference intervals. Results were used to evaluate for hypopituitarism. For participants undergoing replacement therapy at the time of the baseline visit, pre‐therapy data were used to confirm deficiencies.

Criteria for hypopituitarism included secondary adrenal insufficiency (AI): morning cortisol level ≤3 μg/dL or peak cortisol after cortrosyn stimulation <18 μg/dL; secondary hypothyroidism with free thyroxine <0.70 ng/dL (in our laboratory) with a normal or low thyroid‐stimulating hormone (normal range 0.35–4.94μIU/mL) and no history of primary hypothyroidism; secondary hypogonadism with male testosterone level <280 ng/dL with normal or low LH/FSH; premenopausal females with amenorrhea with normal or low gonadotropins and low oestradiol; and postmenopausal females with gonadotropins below menopausal range. As previously considered by other authors,^9^ IGF‐1 levels were characterized as normal or low, that is to say, below the lower limit of normal for age. Patients were categorized as being hypopituitary if they had a deficiency of one or more pituitary axes.

The suspected pituitary adenomas were best visualized on the T1‐weighted coronal and sagittal pre‐ and post‐contrast images. Lesion size was measured directly from the T1‐weighted post contrast images. T2‐weighted images were also used.

By convention, microadenomas measure less than 1 cm and macroadenomas are at least 1 cm in size.^9^

Inclusion criteria included normal prolactin level for lesions <9 mm or a prolactin level <100 ng/mL for lesions ≥10 mm in maximal tumour diameter. We exclude patients without a complete hormonal evaluation.

All tumours that underwent surgical intervention were previously diagnosed by histopathologic analysis, including evaluation of haematoxylin and eosin (H&E) stained slides, periodic acid–Schiff and reticulin histochemical stains and a panel of immunohistochemistry (IHC). The evaluation of the adenohypophyseal hormones expression was performed with following antibodies: GH (polyclonal, Master in vitro), prolactin (clone EP193, Master in vitro), TSH (clone EP254, Master in vitro), ACTH (polyclonal, Master in vitro), FSH (EP257, Master in vitro) and LH (polyclonal, Master in vitro). A subset of tumours were evaluated for the pituitary cell lineage transcription factors, namely (pituitary‐specific transcription factor‐1 PIT‐1/POU1F (polyclonal, Master in vitro), T‐box transcription factor T‐PIT/TBX19 (polyclonal, Master in vitro) and GATA‐3 (clone D13C9, Cell Signalling). Further immunohistochemical study was performed for CK8&18 (B22.1 & B23.1, Cell Marque) and Ki67/MIB‐1 (clone 30‐9, Roche). The MIB‐1 labelling index was reported in hot spots.

### 2.3 Statistical analysis

Continuous variables were summarized as mean and standard deviation (SD), and were compared using the Student's *t*‐test, while categorical variables were described as counts and proportions and were compared using the χ^2^‐test or Fisher's exact test, as appropriate. Cohen's Kappa coefficient was calculated to assess the agreement between biochemical hypopituitarism and hypopituitarism symptoms.

Statistical analysis was performed using SPSS Statistics 27.0 (IBM Corp.). A significance level of 5% was employed.

## 3 RESULTS

The main initial manifestations of the tumours in each group are listed in Table 1. The study sample of patients were mainly composed of men, with a mean (SD) age of 56.8 years (16.7) Table 2 1.

**TABLE 1:** Primary reasons for presentation in incidental and symptoms presentation groups of the cohort.

Incidental	Symptomatic
**Head/neck complaint (*n*)**	**Mass effect (*n*)**
Head injury (8)	Vision‐related (23)
Sinus disease (2)	Headache (12)
Tinnitus (3)	Vision‐related and headache (15)
Hearing loss (1)	**Neurologic (*n*)**
Head pain, transient (4)	Apoplexy symptoms (9)
C‐spine disease (6)	**Pituitary dysfunction**
**Neurologic (*n*)**	Hypopituitarism symptoms (4)
Vertigo (7)	
Dizziness (5)	
Syncope (1)	
AVC or AIT (5)	
Memory complaint (5)	
Seizure disorder (2)	
Trigeminal neuralgia (1)	
Paresthesias (2)	
Brain infection (1)	
**Other (*n*)**	
Panic disease (2)	
Hypertensive crisis (1)	

**TABLE 2:** Incidental versus symptomatic group—comparison.

	Symptomatic (52.9%)	Incidental (47.1%)	*p*‐value
Macroadenoma	91.8%	79.6%	**.060**
Age	56.82	56.73	.976
Sex	M: 54.0% vs. F: 46.0%	F: 53.6% vs. M: 46.4%	.965
Hypopituitarism (analytic)	66.7%	41.1%	.005
Hypogonadism (analytic)	50.8%	23.6%	**.002**
Hypothyroidism (analytic)	46.0%	18.2%	**.001**
GH deficiency (analytic)	50.8%	33.9%	.064
Hypoadrenalism (analytic)	39.7%	14.28%	**.002**
Symptoms of hypopituitarism	27.0%	8.9%	**.011**
Hyperprolactinemia (analytic)	41.3%	32.1%	.303
Symptoms of hyperprolactinemia	22.2%	17.9%	.554
Mass effect symptoms	88.9%	32.1%	**<.001**
Amenorrhea	6.3%	0.0%	.121
Erectile dysfunction	12.7%	5.4%	.168
Surgery	75.8%	50.0%	**.004**
Reintervention (1)	14.9%	3.6%	.245
Radiotherapy	12.9%	7.1%	.301
Null cells	18.8%	36.0%	.242
Gonadotrophinoma	65.6%	44.0%	
Corticotrophinoma	4.2%	10.7%	
Plurihormonal	4.2%	7.1%	
TSH‐pituitary adenoma	2.1%	0%	

*Note*: Symptoms of hypopituitarism: sexual dysfunction/reduced libido, weakness, unintentional weight loss, low blood pressure, cold intolerance, body hair loss and menstrual abnormalities (irregular menses or amenorrhea). The numerator is always the total number of patients, as we only included patients with all hormone measurements. The numerator is always the total number of patients, as we only included patients with all hormone measurements. (1) In patients who were performed first surgery. We highlighted in bold the results with statistical significance.

Participants were then categorized, based on why their pituitary tumour came to medical attention, either to a group of patients whose tumour was incidentally diagnosed (incidental group: 47.1%) or a group whose tumour was symptomatically diagnosed (symptomatic group: 52.9%) Table 2. Tumour‐related complaints that led to diagnosis included signs or symptoms of pituitary dysfunction and those related to mass effect (vision disturbance, headache, neurologic symptoms; Table 1). Headache was included as a tumour‐related symptom, as long as it was not clearly transient or was due to another, clearly identifiable cause. No statistically significant differences concerning age and sex were observed between patients with incidentally or symptomatically diagnosed tumours Table 2.

Hypopituitarism was more common in the symptomatic than in the incidental group (66.7% vs. 41.1%; *p* = .005), as was expected (Table 2). Furthermore, hypopituitarism was more common in males (63.1% vs. 36.9%, *p* = .026) and among patients with macroadenomas (95.2% vs. 4.8%; *p* = .002).

Symptoms of hypopituitarism was higher in the symptomatic group (77.3%) than in the incidental group (22.7%; *p*‐value .011), as was to be expected. Hyperprolactinemia was also more common in the symptomatic group than in the incidental group, despite not being statistically significant (Table 2). Prolactin measurement at the baseline visit found asymptomatic hyperprolactinemia in 21.8% of patients in the cohort overall. No patients were diagnosed as having diabetes insipidus.

Growth hormone deficit was the most common deficiency, which was isolated in 42.9% of patients. Secondary hypothyroidism was isolated in 32.7% of patients, and secondary hypogonadism in 37.8%.

Patients were divided by the presence or absence of one or more symptoms of hypopituitarism (weakness, low blood pressure, body hair loss, unintentional weight loss, cold intolerance, sexual dysfunction/reduced libido and menstrual abnormalities (irregular menses or amenorrhea) within the hypopituitary and eupituitary groups Table 2.

Although patients in the incidental group were diagnosed due to a reason unrelated to the tumour, many of these patients revealed symptoms that were most likely associated with the tumour, when carefully asked.

For example, in the incidental group, 32.1% of participants reported symptoms related to mass effect (visual symptoms or headache), and 22.7% to symptoms of hypopituitarism Table 2.

There is poor agreement between the symptoms of hypopituitarism and the presence of analytic hypopituitarism (*p* = .398).

In both groups, the majority of tumours (87.4%) in our cohort were macroadenomas, especially in the symptomatic group (*p* = .060). As mentioned‐above, hypopituitarism was more frequent among patients with macroadenoma.

75.8% of symptomatically diagnosed patients were submitted to surgery (mostly following the recommendations of the Endocrine Society's guidelines regarding surgical indication) versus 50.0% in the incidental group (*p* = .004). Furthermore, 14.9% of patients in the symptomatic group versus 3.6% of those in the incidental group required at least a second surgery, and 12.9% versus 7.1%, respectively needed radiotherapy, although these results were not significant (*p* = .245 and .301, respectively) Table 2, probably because few patients were re‐intervened as well as undergoing radiotherapy in either group, and therefore the difference was not significant.

Patients with incidental presentation who underwent surgery had cell null tumours in 36.0% of cases, gonadotrophinomas in 44.0% and other histological types (inconclusive, corticotrophinomas or mixed cell) in 20.0% of cases. These results were not significantly different from the results obtained in the symptomatic group of patients (*p* = .242) Table 2.

## 4 DISCUSSION

In this study, which only includes patients with image and endocrine characteristics that were compatible with a NFPA, we observed that many patients had unappreciated signs and symptoms of pituitary disease. More patients in the symptomatic group had hypopituitarism and the majority of patients had macroadenoma. Even patients with microadenoma had hypopituitarism in 18.8% of cases. Usually the hypopituitarism that appears associated with pituitary adenomas is due to the compressive effect of the same on the pituitary gland; however, microadenomas rarely compress normal pituitary gland to cause this dysfunction. Probably not only the size of the tumour but also other individual characteristics of the adenoma and/or the patient (such as genetic alterations or proteomics) could be responsible for these alterations and should be investigated in the near future.

We included only patients with NFPA, as few contemporary studies have evaluated and described the presentation and characteristics of this type of tumour.^9^, ^11^ As our study encompasses both patients with NFPAs undergoing surgical treatment and those managed conservatively, it is a good representation of all the characteristics (clinical and analytical) of this type of tumour nowadays.

In our analysis, we could observe that many of the patients diagnosed with NFPAs in our hospital were diagnosed incidentally (47.1%), which is similar to the results of another recent cohort, which also include surgical and non‐surgical patients with NFPAs. However, this result is much higher than mostly surgical series where the prevalence of incidentally‐diagnosed NFPAs ranged from 9% to 21%.^14^, ^15^, ^16^, ^17^, ^18^

Our definition of incidental diagnosis was similar to the Endocrine Society's guidelines and also similar to that which is employed in most studies,^11^, ^19^ and thus our higher prevalence was probably not due to our definition. We included patients with headaches (one of the most common reasons to perform head imaging studies),^20^, ^21^ in the symptomatic group, although this inclusion is not consensual (many pituitary adenomas can lead to headaches, but it is not always easy to establish a causal relationship between them), but this still lead to a higher percentage of patients who were attributed to symptomatic diagnosis. In other words, this higher prevalence of incidentally diagnosed NFPAs is probably due to the widespread use of computed tomography (CT) and magnetic resonance imaging (MRI) for various clinical diseases.

Furthermore, we observe that 66.7% and 41.1% of patients in the symptomatic and incidental groups, respectively, have hypopituitarism (*p* = .005), and that even among patients with microadenomas, some could have hypopituitarism, which reinforces the importance of also carrying out a hormonal assessment with these patients, in accordance with the Endocrine Society's guidelines, but against others recommendations that also exist.^19^ The prevalence of hypopituitarism in incidentally diagnosed patients is higher than other contemporary series which reported a prevalence of 27.4%,^9^ although we also included only NFPAs, and used a similar definition of hypopituitarism. Furthermore, we observe a prevalence of 95.2% of hypopituitarism in patients with macroadenomas. This discrepancy may be due to the population sample, although both studies have an identical percentage of patients included with macroadenomas. However, taking into account several other studies on NFPAs, partial hypopituitarism could be reported in 37%–85% of patients with NFPAs, which is more similar to our results.^22^, ^23^, ^24^, ^25^ Growth hormone deficiency(GHD) was the most common pituitary deficiency in our cohort, similar to previous reports^8^, ^11^, ^26^; however, GHD cannot be assured, as our participants with low IGF‐1 had a variable number of other hormone deficiencies.^27^, ^28^ Although apoplexy is a rare consequence of pituitary adenomas, we observe nine patients with this type of condition, which may have increased the percentage of cases with some degree of hypopituitarism, as acute hormonal insufficiency is common in this situation and can occur in up to 70% of patients.^29^ NFPAs are the most common pre‐existing adenomas in patients with apoplexy, although this value may be overrepresented, as hormonal hypersecretion/functionality is not evident on evaluation of pituitary adenoma with apoplexy. Partial hypopituitarism develops insidiously and therefore is frequently undetected until the later phase of the disease.

We did not observe any differences between the incidental and symptomatic group in relation to age and sex, which contrast with the results found by Freda al. andOno et al.,^11^ which reported higher age in the incidental group.^9^, ^11^

Patients in the incidental group had a larger percentage of microadenomas than the symptomatic group, probably because as tumour enlarge, they more frequently cause symptoms, as is to be expected. However, many of the incidentally diagnosed patients had large tumours and tumour‐related symptoms when judiciously questioned, which highlights the considerable delay in their diagnosis. In fact, the signs and symptoms of pituitary disease are often unrecognized by the patient and/or the physician; however, a greater degree of awareness about this condition could help avoid more harmful consequences for patients that arise as the tumour progresses. The percentage of incidental tumours that were macroadenomas in our series is much higher than reported in other series^10^; however, the reason for this cannot be clarified through the collected data and needs further research.

We found that 41.3% and 32.1% of patients with incidentally and symptomatically diagnosed NFPAs, respectively, had high prolactin levels on analytic evaluation, without significant differences between groups. Although more patients in the symptomatic group had macroadenomas, this could be due to the high prevalence in the incidental group, and also because hyperprolactinemia is commonly encountered due to the stalk effect.^30^

No patient had central diabetes insipidus, as this pathology is rare in this context and should lead to suspicious of craniopharyngioma, hypophysitis or metastasis.^31^

Surgery is the first choice of treatment in those patients with NFPAs that have symptoms related to tumour mass effect, and should be considered in those with hormonal deficiencies.^13^, ^32^ In patients with apoplexy, surgery is only recommended in patients with visual deficits and deterioration of consciousness.^33^, ^34^ Surgical intervention was required in a higher percentage of patients diagnosed with symptoms, probably because they were also the patients with the highest percentage of macroadenomas. Radiotherapy should be considered in patients with cavernous sinus involvement that is not adequate for surgical removal, or in significant post‐operative residual tumours.^13^ Furthermore, symptomatic patients with incomplete resection or recurrence after surgery should undergo a second surgery and/or radiotherapy, although in our study the differences were not statistically significant, probably this was a result of there being few patients who required surgical re‐intervention and radiotherapy. Previous studies reported that patients with pituitary incidentalomas (irrespective of hormonal hypersecretion) could have a better postoperative clinical outcome than non‐pituitary incidentaloma,^10^, ^11^ but there is a lack of such data, and as far as the authors know, there are still few studies exclusively evaluating adenomas.

With regards to the histological type of tumour among the operated patients, there were no significant differences between the two groups. The majority of tumours (44.0% in the incidental group and 65.6% in the symptomatic group) were gonadotrophinomas, which is line with previously existing results,^35^ which also reveals a predominance of gonadotrophinomas among patients with nonfunctioning pituitary adenomas.

As far as the authors know, this was the first study in surgical and non‐surgical series to be carried out that compare the histological type of ANF (among patients in the incidental and symptomatic groups); however, larger samples may be needed to draw more reliable conclusions.

Our study has some limitations. The data were retrospectively collected, and thus hormonal assessment of pituitary function, tumour size and the radiological characteristics of some patients were not available and were initially excluded. We only assess the differences regarding the type of treatment, and thus it would be important to assess the behaviour of these tumours in the long term, in order to draw more conclusions about the differences in their prognosis. Furthermore, our classification of null cell adenomas was based on negative results of all pituitary hormone on immunohistochemistry; however, the 2017 WHO classification of pituitary adenomas state that the majority of NFPA negative for gonadotrophin staining are, indeed, gonadotroph adenomas when the SF‐1 transcription factor is used and the results may become a little different when analysing this marker.

Additionally, many patients with normal IGF‐1 levels can have GH deficiency if appropriate stimulation tests are used, therefore, although GH deficit was the most common deficit, the results may have been underestimated. We considered as GH deficiency if several other pituitary deficiencies were present. Even so, the GH deficit was the most commonly detected deficit, which is in line with what is already known that when a pituitary deficit begins to appear, it begins to arise from the deficit of GH secretion.^36^ We considered as GH deficiency if several other pituitary deficiencies were present.

Our study also has several strengths, such as the fact that it is the first study in surgical and non‐surgical series to compare nonfunctioning adenomas, not only in terms of their clinical and analytical characteristics, but also in terms of the treatment and pathology type of the tumour. In addition, it includes a large sample of patients which mostly represents the general population, for even though our hospital is a reference centre in terms of the surgical management of pituitary tumours, only about 30% of patients were referred by neurosurgeons.

In summary, the aim of our study was to highlight the increasing prevalence of incidentally diagnosed nonfunctioning adenomas, as well as the need for more clinical suspicion for this condition, as many of the patients manifesting this type of diagnosis already present previously unknown symptoms related to the tumour. It is also intended to highlight the need for hormonal assessment in all the tumours, both the incidentally and symptomatically diagnosed ones, be they macro or microadenomas. It would only be possible to understand the behaviour of these tumours better and act accordingly if contemporary cohorts including patients undergoing or not undergoing surgical intervention were studied. Further studies can clarify the differences between these types of tumours better, especially with regards their long‐term prognosis.

## AUTHOR CONTRIBUTIONS

Vanessa Guerreiro: conceptualization (lead), writing—original draft, investigation; Fermando Mendonça: investigation, writing review and editing (equal); Paula Freitas: writing review and editing (equal); Josué Pereira: writing review and editing (equal); Irene Bernardes: reviewed the article; Jorge Pinheiro: writing review and editing (equal); Tiago Guimarães: writing review and editing (equal) Davide Carvalho: writing review and editing (equal).

## CONFLICT OF INTEREST STATEMENT

The authors declare that they have no conflict of interest.

## ETHICS STATEMENT

All procedures performed in studies involving human participants were in accordance with the ethical standards of the national guidelines.

## PATIENT CONSENT STATEMENT

For this type of study formal consent is not required in accordance with the national legislation and the institutional requirements.

## Data Availability

The data sets used and/or analysed during the current study are available from the corresponding author on reasonable request.
